# Cacodylic Acid

**Published:** 1901-04-13

**Authors:** 


### CACODYLIC ACID.

While on the question of arsenic it may be well to refer to some remarks made by Mr. Hutchinson in this month's *Polyclinic.* Speaking of the claims made for cacodylic acid, to the effect, namely, that it may be administered in large doses without disagreement and that it does not, like other preparations of arsenic, cause discoloration of the skin or peripheral neuritis, he says that it may be the fact that we really want a drug which is capable of producing these very effects. What we want to know is not that a considerable dose of arsenic can be given, but that the disease will be cured. The good effects of cacodylate of sodium as vaunted are, he says, precisely those which we have hitherto attributed to the older preparations of arsenic, but we must not assume that because a poison can be given in larger quantities in one salt than it can in another, therefore the one best tolerated is the better remedy; it is possible that it may be simply the less efficient.

